# Early social communication and language development in moderate-to-late preterm infants: a longitudinal study

**DOI:** 10.3389/fpsyg.2025.1556416

**Published:** 2025-04-03

**Authors:** Blanca Palomero-Sierra, Victoria Sánchez-Gómez, María Magán-Maganto, Álvaro Bejarano-Martín, Irene Ruiz-Ayúcar, Victoria B. de Vena-Díez, Giselle V. Mannarino, Emiliano Díez-Villoria, Ricardo Canal-Bedia

**Affiliations:** ^1^Institute for Community Inclusion (INICO), University of Salamanca, Salamanca, Spain; ^2^Institute for Biomedical Research of Salamanca (IBSAL), University of Salamanca, Salamanca, Spain; ^3^Department of Basic Psychology, Psychobiology, and Behavioral Science Methodology, University of Salamanca, Salamanca, Spain; ^4^Department of Personality, Assessment, and Psychological Treatments, University of Salamanca, Salamanca, Spain; ^5^University Hospital of Salamanca, Salamanca, Spain

**Keywords:** moderate-to-late preterm infants, prematurity, social communication, language development, cognitive development, neurodevelopment, early predictors, longitudinal study

## Abstract

This study investigates early development and language acquisition in moderate-to-late preterm (MLPT) infants, focusing on social communication as a key factor. Using a longitudinal design, social communicative, cognitive and language outcomes were assessed at 12, 18, and 24 months in 106 infants, including 49 MLPT and 57 full-term (FT) infants. Standardized tools, including the Bayley Scales of Infant and Toddler Development (Bayley-III), the Vineland Adaptive Behavior Scales (Vineland-3), and the Social Attention and Communication Surveillance-Revised (SACS-R), were used to assess early developmental performance. Group differences and the interaction between group and assessment time points were analyzed to examine developmental patterns over time. Additionally, predictive models identified early indicators of receptive and expressive language performance at 24 months. The results revealed significant developmental delays in the MLPT group compared to their FT peers, with receptive language showing the most pronounced deficits. Early social communication behaviors, such as pointing, following a point, and attending to sounds at 12 months, emerged as strong predictors of both receptive and expressive language performance. Cognitive abilities also played a significant role, particularly in receptive language development. These findings underscore the utility of tools like the SACS-R in identifying early communication challenges and guiding tailored support strategies. Sustained developmental monitoring and targeted interventions that foster communication skills may promote positive language outcomes in MLPT infants, supporting their long-term developmental potential within this population with increased developmental needs.

## 1 Introduction

Early social communication constitutes a prerequisite for both receptive and expressive language development, as it helps infants understand the purpose and function of language in social contexts, as well as in the acquisition of formal aspects of language (Bejarano-Martín et al., 2020; Määttä et al., 2012; Tomasello, 1992). Social communication refers to the ability to use verbal and nonverbal cues to interact with others in different situations. These early cues—such as gestures, facial expressions, vocalizations, word production, and joint attention—are socially oriented. They reflect a natural tendency to understand and share interests, experiences, and emotions, as well as to engage in interpersonal interactions (Hansen et al., 2018; Jethava et al., 2022). If these cues are absent or lack social intent, it may indicate developmental concerns and potential challenges in language acquisition. For example, the absence or delay of spoken language by age two is one of the earliest and most prominent warning signs of neurodevelopmental conditions, such as autism; however, these delays are often preceded by a paucity or lack of earlier social communication behaviors (Barbaro and Dissanayake, 2010; Delehanty et al., 2018; Nitzan et al., 2023). Additionally, early social communication is closely linked to cognitive development. The ability to share attention, recognize social cues, and respond appropriately shapes how a child processes social information and interacts with the environment (Jethava et al., 2022; Delehanty et al., 2018). Sharing attention with a social partner is considered a key milestone in infant neurodevelopment, as it promotes social learning opportunities and supports language acquisition (Olafsen et al., 2012; Tomasello, 2006). Consequently, early assessments of social and attentional communication are crucial for identifying the likelihood of diverse neurodevelopmental trajectories. The relationship between early social communication, language and cognitive development is bidirectional and complex, with each domain influencing the other rather than following a simple causal pathway.

Many external factors can influence neurodevelopment. For example, prematurity can significantly impact multiple domains, increasing the likelihood of neurodevelopmental disorders (Mateus et al., 2019; Pettinger et al., 2023). Significant developmental challenges are often attributed to infants classified as very preterm (Gestational age [GA] between week 28 and week 31 + 6 days) or extremely low preterm (less than 28 weeks of GA). However, there has been a recent increase in research focusing on moderate-to-late preterm infants (MLPT, GA between week 32 and week 36 + 6 days). As the largest group of preterm infants, MLPT infants have been increasingly suggested to be at a relatively higher risk of neurodevelopmental challenges compared to full-term (FT) infants (Pettinger et al., 2023). Likewise, these infants are also considered physiologically and metabolically immature (Mitha et al., 2024; Palumbi et al., 2018). The final weeks of gestation are crucial for neurobiological development, supporting the maturation of neural circuits and structural and functional connectivity involved in attention, sensory integration, emotional regulation, and higher-order cognitive functions (La Rosa et al., 2024; Tau and Peterson, 2010; Leisman et al., 2024). For example, when these processes are disrupted after week 32, MLPT infants may present, among other challenges, an underdeveloped sensory system (La Rosa et al., 2024). This can affect their ability to process tactile stimuli effectively, which is particularly significant given that early parent-infant interactions rely on touch—such as maternal skin-to-skin contact—to provide comfort, promote a sense of security, and facilitate subsequent socio communicative and emotional development (La Rosa et al., 2024). Considering these neurobiological mechanisms, a better understanding of their impact on MLPT infants is essential. Cognitive impairments have been identified as the most common adverse outcome in MLPT, followed by neuromotor and sensory challenges, as well as neurodevelopmental conditions (Palumbi et al., 2018; Johnson et al., 2015). Given the interdependence of developmental domains, it is crucial to identify and address social communication challenges as an early stage (Jethava et al., 2022; De Schuymer et al., 2011). Early interventions may foster not only cognitive and social development but also improvements in receptive and expressive language skills (Bejarano-Martín et al., 2020; Olafsen et al., 2012).

In the literature, most studies comparing preterm and FT infants focus on extremely and very preterm groups (De Schuymer et al., 2011; De Schuymer et al., 2012; Benassi et al., 2016; Evrard et al., 2011; Olafsen et al., 2006; Sansavini et al., 2015; Muller-Nix et al., 2004; De Groote et al., 2006), with fewer examining MLPT specifically or including all preterm categories (Mateus et al., 2019; Pettinger et al., 2023; Mitha et al., 2024; De Schuymer et al., 2012; Montirosso et al., 2010). However, these studies report that preterm infants, regardless of category, exhibit lower performance in early social communication behaviors compared to their FT peers. At 12 months, infants typically develop joint attention and basic communicative gestures, which are foundational for subsequent language acquisition. Preterm infants show reduced social response, fewer initiating behaviors, gestures, and vocalizations (Mateus et al., 2019; De Schuymer et al., 2011; Benassi et al., 2016; Evrard et al., 2011; Olafsen et al., 2006). They also demonstrate less engagement in interactions and a greater tendency for escape behaviors (De Schuymer et al., 2012; Evrard et al., 2011; Sansavini et al., 2015; Montirosso et al., 2010). By 18 months, rapid vocabulary growth and increased complexity in social interactions occur, making it a crucial period for identifying persistent delays and intervening accordingly. At this point, preterm infants continue to exhibit significantly reduced social responsiveness and initiating behaviors, often displaying greater passivity during interactions (Mateus et al., 2019; De Schuymer et al., 2011; Muller-Nix et al., 2004). At 24 months, language abilities are more pronounced, and infants are expected to have an increasing expressive vocabulary and more sophisticated receptive language skills. However, preterm infants demonstrate significantly less communicative behaviors and interest in interactions, including reduced social initiation (De Groote et al., 2006). Consequently, delays in language abilities become apparent at this age, with preterm infants showing difficulties in both language comprehension and production (Sansavini et al., 2015). Beyond infancy, language challenges in preterm children can persist into school age and adolescence (Palumbi et al., 2018), particularly in grammatical and morphosyntactic skills, narrative abilities, and vocabulary development. These children often struggle to construct complex sentences, organize coherent stories, and demonstrate reduced expressive and receptive vocabulary (Crosbie et al., 2010; Guarini et al., 2016; Van Noort-van Der Spek et al., 2012; Zimmerman, 2018). Conversational difficulties include reduced initiative and challenges in maintaining reciprocal interactions, thereby leading to comprehension delays that may impact their ability to follow complex instructions and engage in advanced linguistic tasks (Zimmerman, 2018; Martínez-Nadal and Bosch, 2020; Sanchez et al., 2020).

These early difficulties in social communication are observable as early as the first year of life in preterm infants (Pérez-Pereira et al., 2013). However, the extent and significance of such differences compared to FT peers can vary, influenced by factors such as GA, birth weight, perinatal or clinical characteristics, and variability in assessment methods (Pérez-Pereira, 2021; Sansavini et al., 2011). These behaviors are typically assessed through observations of mother-infant interactions, with researchers often developing coding schemes for these dyadic observations; nevertheless, standardized tools such as the Early Social Communication Scales (ESCS) (Mundy et al., 2003) are also frequently used. Previous research has examined how preverbal skills, assessed using the ESCS within the first 2 years of life, may mediate the relationship between preterm birth and later language acquisition (De Schuymer et al., 2011). To our knowledge, there is a scarcity of studies specifically addressing the social communication development of MLPT infants during the first 2 years of life. Addressing this gap is crucial, as these infants may experience subtle yet meaningful developmental challenges that can shape later language acquisition and broader socio-cognitive outcomes, highlighting the need for early identification and targeted interventions. The objectives of this study were therefore twofold: (i) to compare early developmental outcomes between MLPT and FT infants at 12, 18, and 24 months, examining the interaction between group and assessment time points; and (ii) to identify which social communication behaviors observed at 12 months, along with clinical, sociodemographic, and other relevant developmental measures, are the strongest predictors of language outcomes at 24 months, both within the MLPT preterm group and across a combined sample of preterm and FT infants. The hypotheses were, for objective one, MLPT preterm infants would exhibit significant differences in early developmental outcomes compared to FT infants at the assessment time points, and that a significant interaction between group and assessment time points would be observed, suggesting that the developmental trajectories of the two groups differ over time. For objective two, it was hypothesized that social communication behaviors observed at 12 months, along with clinical factors and sociodemographic variables, would be significant predictors of language outcomes at 24 months. Additionally, within the combined sample, prematurity would emerge as a significant predictor of language outcomes at 24 months.

## 2 Method

### 2.1 Participants

A total of 106 infants participated in this study (49 MLPT, 57 FT), with birth dates ranging from June 2020 to August 2023. The findings reported are part of a longitudinal study conducted by the University of Salamanca in collaboration with the University Clinical Hospital of Salamanca, Spain. The original cohort was assessed at 12, 18, 24, and 36 months. MLPT infants were recruited from the neonatal intensive care unit (NICU). The inclusion criteria were as follows: (i) GA less than 37 weeks; (ii) normal neuropediatric status, with no evidence of brain injury, syndromes, or congenital malformations; (iii) absence of hearing or visual impairments; and (iv) no siblings diagnosed with a neurodevelopmental condition. Infants requiring stabilization and NICU admission after birth were included, provided they met the other inclusion criteria. Healthy FT infants (GA > 37 weeks) were recruited from health centers in the provinces of Salamanca, Zamora, and Valladolid, Spain.

The composition of the sample is summarized in Table 1. The minimum sample size was determined based on prior studies using similar methodologies and tools (De Schuymer et al., 2011; Olafsen et al., 2006), and was exceeded in this study. Additionally, a *post hoc* power analysis indicated that our minimum final sample size per group (*n* = 41) provided 88% power to detect an effect size of *d* = 0.7 at a significance level of 5%, with reference studies reporting effect sizes ranging from medium to large. The two groups (MLPT/FT) showed significant differences in GA and birth weight. Both groups were equivalent in terms of gender distribution, parental age at conception, and corrected age at each assessment point. However, the MLPT group showed a significantly higher incidence of twin pregnancies, as well as pregnancy-related and neonatal complications. Significant differences were also found in family socioeconomic status (SES), with the MLPT group exhibiting a lower SES compared to the FT group. A considerable proportion of participants in both groups scored below the normative threshold (standard score < 85) on the Bayley-III cognitive and language scales, as well as on the Vineland-3 Adaptive Behavior Composite (ABC).

**Table 1:** Sample composition and group comparisons on key sociodemographic and neonatal variables.

	MLPT	FT	*p*
GA, M (SD)	34.51 (1.91)	39.72 (1.221)	<0.001**
Birth weight, M (SD)	2337.35 (539.57)	3256.14 (401.85)	<0.001**
< 1,500 g^a^	6.12	0	0.058
Male^a^	65.30	54.38	0.254
IVF^a^	12.24	8.77	0.647
Twin pregnancy^a^	12.24	0	0.007**
Pregnancy-related complications^a^	61.22	33.33	0.010*
Cesarean^a^	34.69	15.78	0.144
Stay in NICU^a^	57.14	10.53	<0.001**
Neonatal complications^a^	67.34	42.11	0.025*
Fetal distress	32.65	32.08	
Hypoglycemia	16.33	0	
Neonatal respiratory disorders	32.65	3.77	
Intraventricular hemorrhage (grade I)	2.04	0	
Retinopathy of prematurity (grade I)	2.04	0	
Mother’s age at conception, M (SD)	35.41 (4.14)	35.41 (3.61)	0.819
Father’s age at conception, M (SD)	37.55 (5.41)	38.37 (5.23)	0.472
SES, M (SD)	36.89 (10.86)	43.73 (10.22)	0.003**
Working class^a^	20.41	9.75	
Lower-middle class^a^	38.78	20.83	
Upper-middle class^a^	36.73	46.34	
Upper class^a^	4.08	17.07	
Age at 12 months assessment (months)^b^, M (SD)	11.92 (0.756)	11.89 (0.658)	0.844
Age at 18 months assessment (months)^b^, M (SD)	18.14 (0.878)	17.90 (0.823)	0.180
Age at 24 months assessment (months)^b^, M (SD)	24.34 (0.761)	23.88 (0.761)	0.055

	12 months		18 months		24 months	
	MLPT	FT	MLPT	FT	MLPT	FT
Bayley-III						
Cognitive scale (%)^c^	2.08	0	9.09	6	9.76	4.17
Language scale (%)^c^	12.5	10.91	36.36	14	31.71	14.58
Vineland-3						
ABC (%)^c^	6.25	5.56	18.60	10	38.46	14.58

This table summarizes the sample composition and compares MLPT and FT groups in GA, birth weight, SES, and other clinical variables. MLPT, Moderate-to-late preterm; FT, Full-term; SES, Family socioeconomic status. ** *p* <0.01; *p < 0.05 (Bilateral). ^a^Percentage. ^b^Corrected for preterm. ^c^Standard score < 85.

### 2.2 Procedure

The study was reviewed and approved by the Research Ethics Committee of the University of Salamanca (Registration Number: 562_211220), in full compliance with all ethical standards. Informed consent was obtained from all participating families. Families of the MLPT infants were contacted by phone through the University Clinical Hospital of Salamanca, while families of FT infants were invited to participate during their 9-month pediatric check-up at health centers. Interested families filled out a form that pediatricians forwarded to the research unit. Both groups were invited to participate in a neurodevelopmental evaluation, and appointments were scheduled for 12, 18, and 24 months, with a margin of 1 month. Corrected age was systematically applied to the MLPT cohort at each assessment point. All assessments were conducted in a single session, with duration varying based on the assessment time point (12, 18, or 24 months) but never exceeding 2 h. Sessions took place in a child-friendly environment designed to minimize distractions, with two trained examiners, the infant, and at least one parent present. Examiners were blinded to GA and clinical history, although complete blinding to group status was not always possible. Each session began with an informal conversation with the parent to explain the procedure and their role. The Bayley-III was administered first, starting with the cognitive scale, followed by the language scale, and concluding with the SACS-R. Meanwhile, one of the parents, seated nearby, completed the Vineland-3 reported form on a tablet. At 12 months. After these assessments, a pediatric neurologist conducted a physical examination and an interview to collect clinical and sociodemographic information.

### 2.3 Measures

#### 2.3.1 Clinical and sociodemographic variables

Several variables related to pregnancy and childbirth were considered, including GA calculated in complete weeks from the mother’s last menstrual period, birth weight, twin pregnancy, method of conception (e.g., *in vitro* fertilization [IVF]), pregnancy-related complications (e.g., gestational diabetes, hypertension, hypothyroidism), mode of delivery, and length of stay in the NICU. Neonatal complications were also recorded, such as fetal distress; hypoglycemia; neonatal respiratory disorders (including respiratory distress syndrome and apnea); intraventricular hemorrhage (grade I); and retinopathy of prematurity (grade I). Additionally, parental age at conception was recorded, and SES was determined using the Hollingshead Four-Factor Index of Social Status (Hollingshead, 1975), which evaluates SES based on the highest educational attainment and employment status of both parents.

#### 2.3.2 Bayley scales of infant and toddler development—third edition (Bayley-III)

The Bayley-III is a widely used instrument for assessing cognitive, language, and motor development in infants and toddlers up to 42 months, and has demonstrated validity and strong reliability in preterm populations (Aylward, 2017). In this study, its standardized Spanish version (Bayley, 2015) was used by the examiners to assess cognitive and language development. Raw scores were converted into scaled scores (range 1–19), and then into composite scores (mean = 100, SD = 15). Composite scores were used descriptively for the cognitive and the language scale, which combines the receptive and expressive subscales. For the primary analyses, scaled scores were used, resulting in three outcome measures: Cognitive, Receptive, and Expressive.

#### 2.3.3 Vineland adaptive behavior scales—third edition (Vineland-3)

Social-adaptive development was assessed using the Spanish version of the Vineland-3 comprehensive parent-caregiver form (Sparrow et al., 2016), based on parent-reported information. Scaled scores (range 1–24) were derived for each subdomain, along with ABC, a composite score that reflects overall adaptive behavior (*M* = 100, SD = 15). Seven outcomes were derived: Receptive language, Expressive language, Personal, Interpersonal Relationships, Play and Leisure, and ABC.

#### 2.3.4 Social attention and communication surveillance (SACS-R)

Social attention and communication were evaluated using the SACS-R, a valid and reliable screening tool designed for identifying behaviors related to social attention and communication difficulties in children aged 12–36 months (Barbaro and Dissanayake, 2010). It was originally developed to detect early signs of autism and is widely recognized as one of the most widely used tools. However, it has also been shown to be effective in identifying developmental and language delays in children (Barbaro et al., 2022). The tool includes age-specific critical behavioral items, which are positively scored when observed by the examiner (e.g., pointing, waving “bye-bye,” imitation or response to name). The absence of these behaviors may indicate potential challenges in social attention and communication difficulties. No modifications were made to the scale. Eleven outcomes from the 12-month assessment were analyzed, reflecting key early social communication behaviors (see Table 2).

**Table 2:** Descriptive statistics for MLPT and FT groups across assessment points.

Outcomes	12 months			18 months			24 months		
Bayley-III^a^ (Observed)	MLPT	FT	*p* (*d*)	MLPT	FT	*p* (*d*)	MLPT	FT	*p* (*d*)
Cognitive	10.73 (1.84)	11.73 (2.20)	0.015* (0.490)	10.48 (2.92)	11.48 (3.98)	0.110	10.83 (3.46)	12.92 (3.81)	0.009** (0.571)
Receptive	8.56 (1.89)	9.16 (2.00)	0.121	8.07 (2.04)	9.96 (2.46)	<0.001** (0.833)	8.41 (2.09)	10.35 (2.47)	<0.001** (0.843)
Expressive	9.00 (1.41)	9.36 (1.67)	0.240	7.86 (2.23)	8.44 (1.72)	0.162	7.68 (2.53)	9.25 (2.86)	0.008** (0.577)

Vineland-3^a^ (Reported)	MLPT	FT	*p*	MLPT	FT	*p (d)*	MLPT	FT	*p*
Receptive language	14.60 (1.77)	14.69 (1.79)	0.819	14.44 (2.84)	14.84 (2.20)	0.444	14.38 (4.42)	15.13 (2.75)	0.365
Expressive language	14.00 (1.75)	13.78 (1.81)	0.532	12.88 (2.28)	13.46 (2.38)	0.239	12.92 (3.70)	13.94 (2.71)	0.144
Personal	15.31 (2.03)	15.07 (1.62)	0.512	14.28 (2.19)	14.94 (1.98)	0.130	13.46 (2.63)	13.65 (2.10)	0.718
Interpersonal relationships	14.58 (1.33)	14.76 (1.24)	0.492	13.51 (1.61)	14.02 (1.67)	0.140	14.31 (5.43)	14.04 (1.75)	0.770
Play and leisure	13.96 (1.24)	14.06 (1.86)	0.800	13.40 (2.47)	14.22 (2.03)	0.081	13.64 (3.30)	13.73 (2.34)	0.889
ABC	97.87 (8.38)	98.69 (9.2)	0.640	94.58 (10.43)	97.82 (9.38)	0.118	91.44 (16.36)	94.29 (10.67)	0.351

SACS-R^b^ 12 months (Observed)	MLPT	FT	*p*
Pointing	0.367	0.382	0.879
Eye contact	0.020	0.018	0.934
Waving ‘bye-bye’	0.429	0.327	0.287
Response to name	0.020	0.000	0.287
Imitation	0.184	0.091	0.166
Follows point	0.041	0.06	0.906
Social smile	0.020	0.036	0.627
Conversational babble	0.061	0.018	0.255
Says 1–3 clear words	0.367	0.509	0.146
Understands simple instructions	0.265	0.182	0.306
Attending to sounds	0.041	0.018	0.491

This table presents means and standard deviations for Bayley-III and Vineland-3 measures for MLPT and FT groups at each assessment point (12, 18, and 24 months), and SACS-R outcomes at 12 months. Significant differences between groups are indicated along with corresponding *p*-values and effect sizes (Cohen’s *d*). MLPT, moderate-to-late preterm; FT = Full-term. ** *p* < 0.01; * *p* < 0.05 (Bilateral). ^a^ Means and standard deviation, means comparison’ *p* value and Cohen’s *d* are reported (*d*). ^b^ Proportions (risk/total) rather than means are reported. Chi-squared tests were performed and obtained *p* value are reported.

### 2.4 Analysis

Prior to conducting the analyses, variability related to GA within the preterm group was partially controlled by restricting the analysis to MLPT infants, thereby reducing the range of GA. However, four infants outside the MLPT category were included, as their presence did not significantly impact the results. Descriptive statistics were computed for all demographic and clinical variables, and group differences were examined using t-tests for continuous variables and Chi-square tests for categorical variables.

To address the first aim —comparing early developmental outcomes between MLPT and FT infants across assessment points— *t-*tests were performed, with Cohen’s *d* reported as the effect size for significant findings. For categorical variables, Chi-square tests were conducted. Variables showing significant group differences were further analyzed using a 2 × 3 repeated-measures factorial ANOVA to examine main effects and interaction effects. Partial eta squared (*η^2^p*) was used to quantify effect sizes for overall model, while Cohen’s *d* was calculated for *post hoc* contrasts. When the assumption of sphericity was violated, the Huynh-Feldt correction was used. Bonferroni post hoc tests were applied to adjust for multiple comparisons.

To address the second aim—identifying predictors of language outcomes at 24 months— multiple linear regression models were employed. Key predictors were initially identified through bivariate correlations of clinical data and assessment outcomes collected at 12 months, with language outcomes at 24 months. Variables with significant correlations were included as independent variables. Predictive models of receptive and expressive language were developed using a stepwise approach to retain the most relevant predictors. Four predictive models were constructed: two global models combining MLPT and FT infants (with prematurity as a variable) and two specific models for the MLPT group (with prematurity as a constant). For each model reported the adjusted R-squared (*R*^2^) (indicating the variance explained, as well as the regression coefficients).

Descriptive analyses, marginal means plots (interaction plots) and multiple linear regression analyses were performed using SPSS v. 28 (IBM Corp, 2021), while factorial ANOVAs were conducted using jamovi (The jamovi project, 2024).

### 2.5 Missing data

Sample sizes varied across measures and visits. Despite attrition at certain assessment points, post hoc power analyses indicated that our study maintained sufficient power to detect meaningful effects. For example, pregnancy and childbirth data were incomplete for four FT participants due to unfinished pediatric examinations. Additionally, 16 FT participants were excluded from the SES index analysis due to a lack of parental employment information. By the 18-month visit, seven participants had withdrawn from the study, and three had missed the visit due to scheduling conflicts. By the 24-month visit, five additional participants had withdrawn. Additional data losses occurred primarily due to infant fatigue during assessments or inaccuracies in parental reporting. As a result, final sample sizes for developmental assessments were as follows: at 12 months, the sample sizes were MLPT = 48 (SACS-R, *n* = 49) and FT = 55 (Vineland-3, *n* = 54); at 18 months, MLPT = 44 (Vineland-3, *n* = 43) and FT = 50; and at 24 months, MLPT = 41 (Vineland-3, *n* = 39) and FT = 48.

## 3 Results

### 3.1 Aim 1

Table 2 summarizes the means and standard deviations for the MLPT and FT groups on the Bayley-III, Vineland-3, and SACS-R measures. At 12 months, significant differences were observed only in Cognitive. By 18 months, significant differences emerged in Receptive. By 24 months, MLPT infants scored significantly lower than their FT peers across all three Bayley-III scales, with moderate to large effect sizes. No significant differences were found in any of the Vineland-3 subdomains at any age or in the SACS-R items at 12 months.

**Figure 1:**
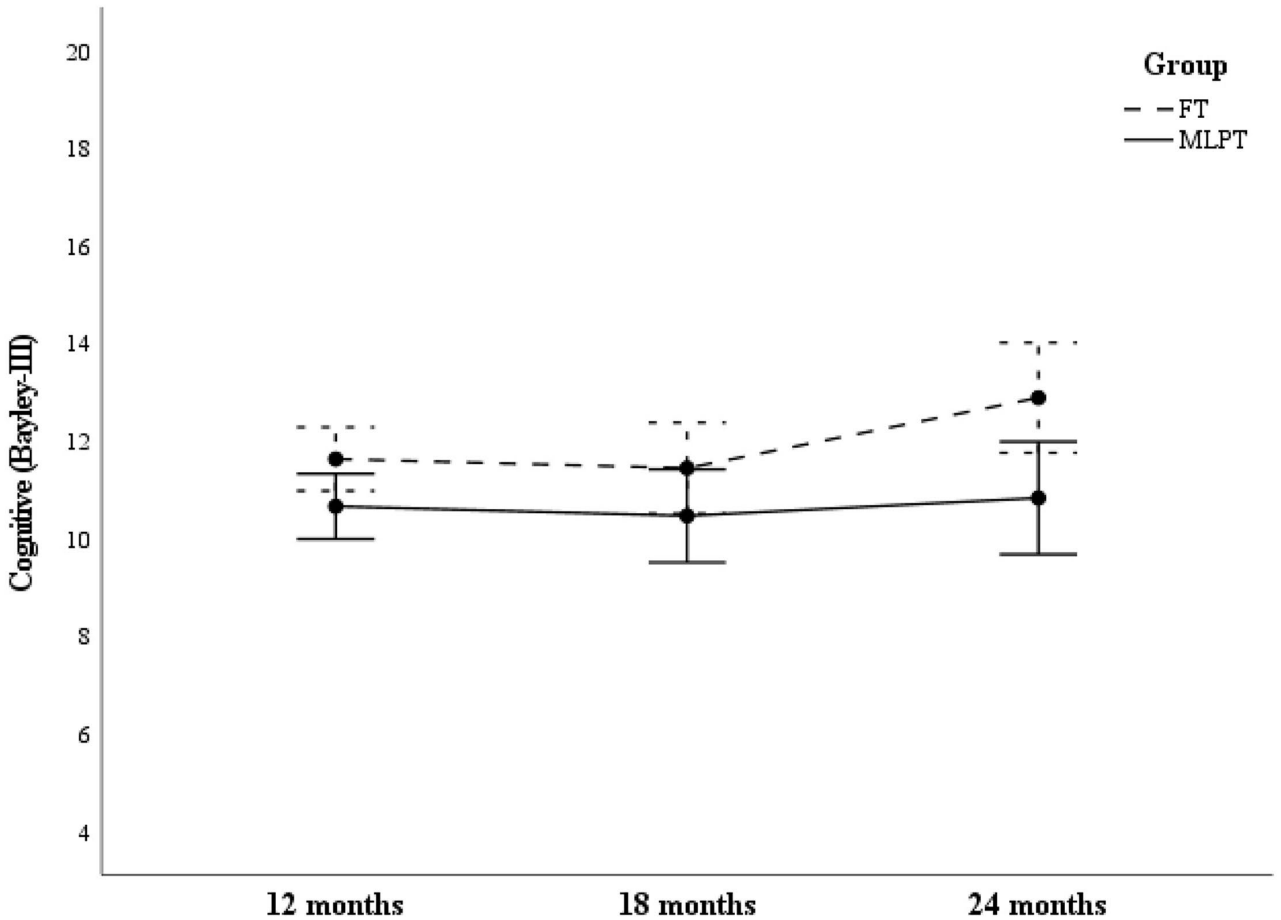
Cognitive scores as a function of group and assessment time point. This figure illustrates differences in Cognitive scores between MLPT and FT groups across assessment points (12, 18, and 24 months).

In the 2 × 3 factorial ANOVA, significant main effects were observed for Cognitive scores, for group (MLPT < FT) (*F* = 5.85, *p* = 0.018, *η^2^p* = 0.067) and assessment time point (*F* = 4.36, *p* = 0.014, *η^2^p* = 0.051). *Post hoc* analysis revealed a significant difference between 18 and 24 months (*t* = −2.822, *p* = 0.018, *d* = 0.31). Although descriptive data suggested a larger group difference at 24 months (Figure 1), no significant interaction effect was observed (*F* = 1.87, *p* = 0.157, *η^2^p* = 0.022).

**Figure 2:**
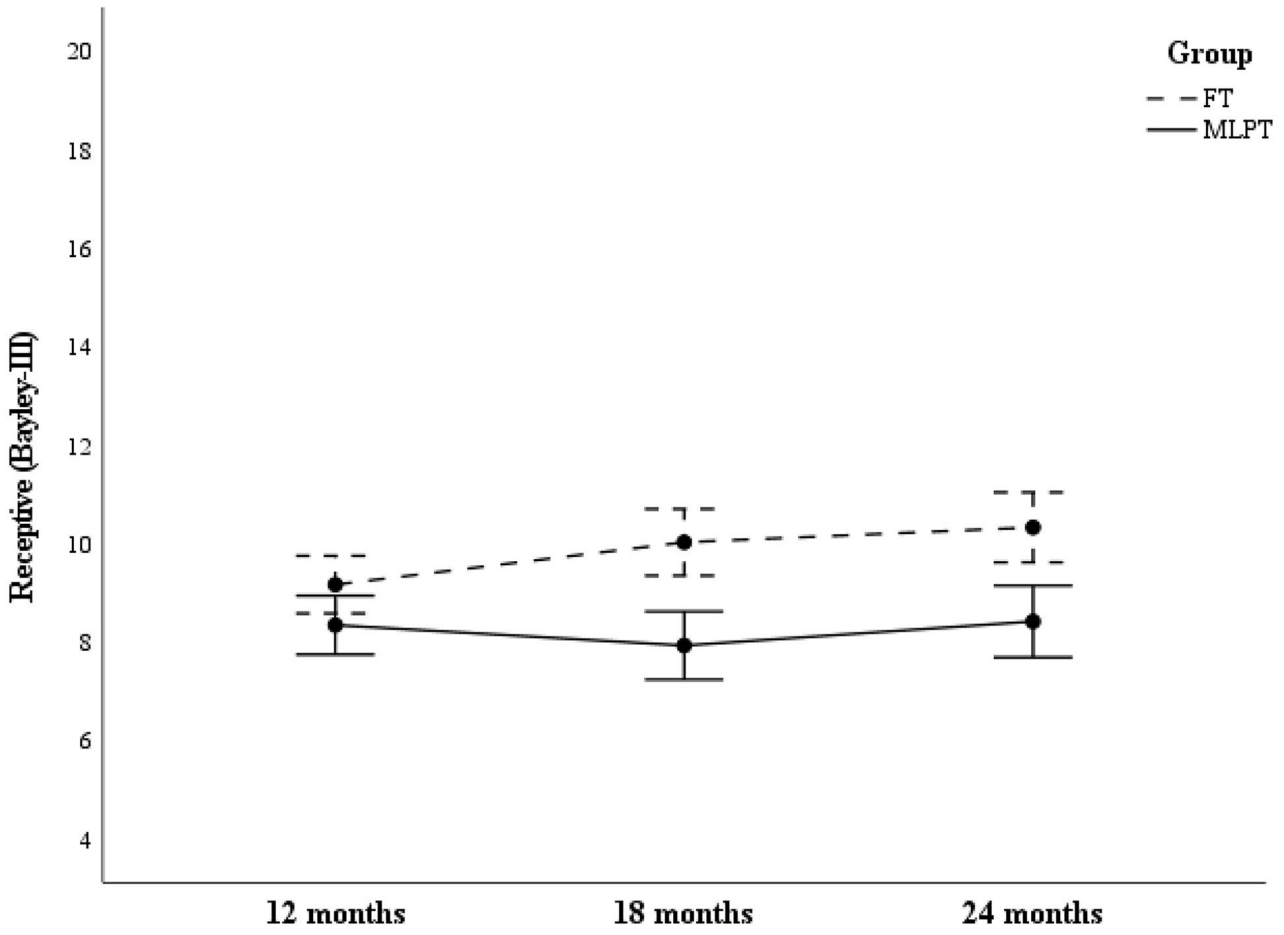
Receptive scores as a function of group and assessment time point. This figure illustrates differences in Receptive scores between MLPT and FT groups across assessment points (12, 18, and 24 months).

For Receptive scores, significant main effects were found for group (*F* = 15.9, *p* < 0.001, *η^2^p* = 0.163) and assessment time point (*F* = 4.20, *p* = 0.017, *η^2^p* = 0.049). Post hoc analysis showed a significant difference between 12 and 24 months (*t* = −2.50, *p = 0.*043, *d* = 0.27). Significant interaction effects were found between group and assessment time points (*F* = 5.09, *p* = 0.007, *η^2^p* = 0.058). As shown in Figure 2, group effects varied by time point, with the interaction ocurring between 12 months (non-significant) and 18 months (significant). Post hoc tests revealed that while no significant differences were observed at 12 months (*t* = 1.96*, p* = 0.806), differences appeared at 18 months (*t* = 4.31, *p* < 0.001, *d* = 0.94) and remained at 24 months (*t =* 0.74, *p* = 0.005, *d* = 0.16).

**Figure 3:**
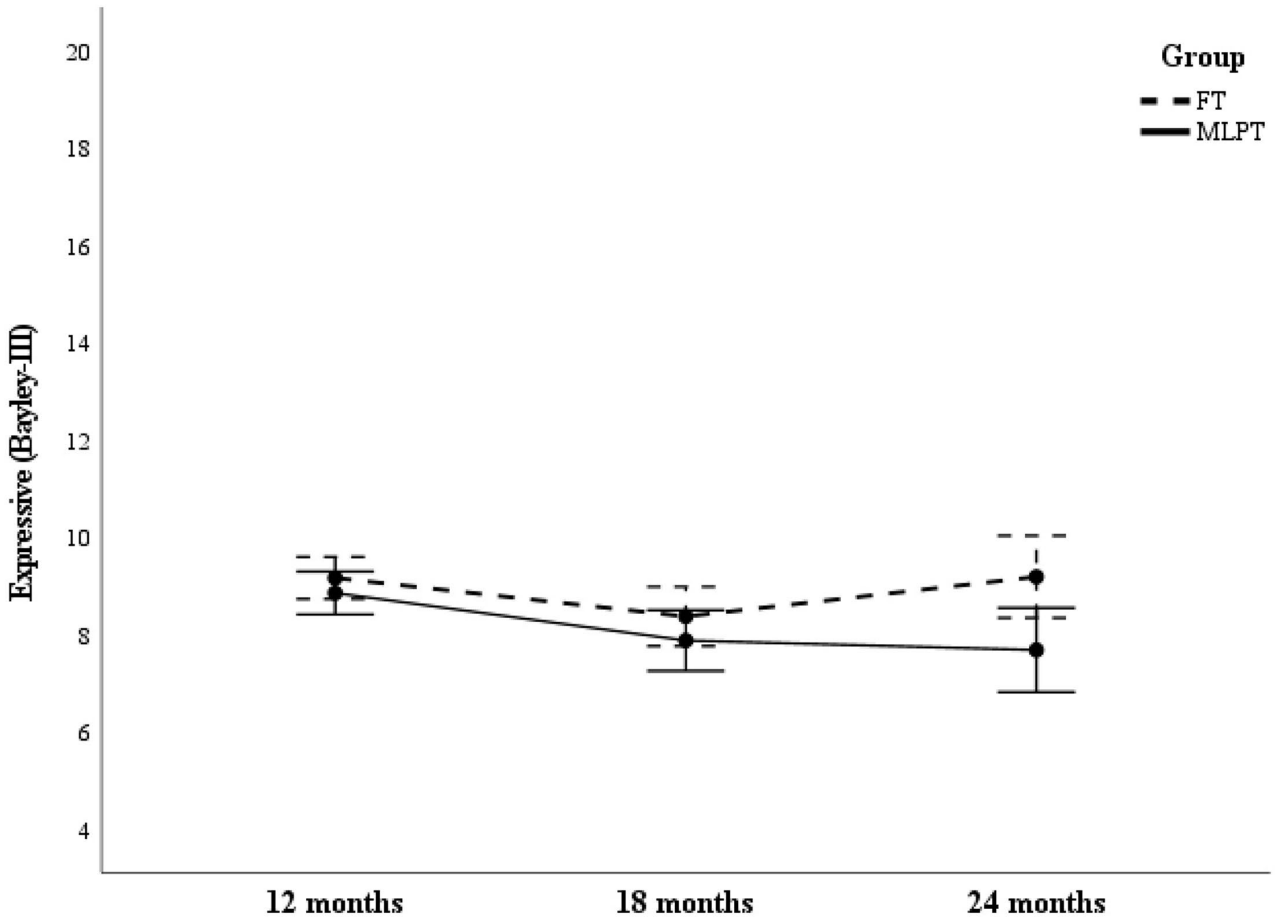
Expressive scores as a function of group and assessment time point. This figure illustrates differences in Expressive scores between MLPT and FT groups across assessment points (12, 18, and 24 months).

Finally, main effects for Expressive scores showed significant differences for group (*F* = 4.379, *p* = 0.039, *η^2^p* = 0.051) and assessment time point (*F* = 6.37, *p* = 0.003, *η^2^p* = 0.072). Post hoc analysis showed significant differences between 12 and 18 months (*t* = 3.91, *p* < 0.001, *d* = 0.43). Significant interaction effects between group and assessment time points approached significance (*F* = 3.28, *p* = 0.046, *η^2^p* = 0.038). Figure 3 shows that group differences varied across time points, with the interaction occurring between 18 and 24 months.

### 3.2 Aim 2

Both global models (including the entire sample of MLPT and FT infants) and specific models (focusing on the MLPT group) were developed to predict language outcomes on the Bayley-III at 24 months. The bivariate correlation matrix, identifying key 12-month predictive variables for the models, is presented in Supplementary Table 1. Table 3 shows the *R*^2^ gain contributed by each variable entered, total adjusted *R*^2^, along with the regression coefficients for each model.

**Table 3:** Regression coefficients and *R*^2^ gains for models predicting 24-month language outcomes.

Model	Dependent variable	Entered variables (12 months)	*R*^2^ change*	Total adjusted *R*^2^	Model coefficients
1	Receptive at 24 months	1. Pointing 2. Cognitive 3. Attending to sounds 4. Follows point 5. Group	*Δ R^2^* = 0.212 *Δ R^2^* = 0.131 *Δ R^2^* = 0.068 *Δ R^2^* = 0.063 *Δ R^2^* = 0.062	*R*^2^ = 0.50	*K* = 7.570 *B_1_* = − 1.338 *B_2_* = 0.275 *B_3_* = − 3.290 *B_4_* = − 2.765 *B_5_* = − 1.204
2	Receptive at 24 months (MLPT infants)	1. Attending to sounds 2. Follows point 3. Cognitive	*Δ R^2^* = 0.310 *Δ R^2^* = 0.226 *Δ R^2^* = 0.100	*R*^2^ = 0.605	*K* = 4.925 *B_1_* = − 4.422 *B_2_* = − 4.920 B_3_ = 0.375
3	Expressive at 24 months	1. Receptive 2. Pointing 3. Interpersonal	*Δ R^2^* = 0.278 *Δ R^2^* = 0.078 *Δ R^2^* = 0.049	*R*^2^ = 0.380	*K =* − 4.399 *B_1_* = 0.568 *B_2_* = − 1.641 *B_3_* = 0.576
4	Expressive at 24 months (MLPT infants)	1. Interpersonal 2. Attending to sounds 3. Receptive	*Δ R^2^* = 0.248 *Δ R^2^* = 0.100 *Δ R^2^* = 0.073	*R*^2^ = 0.372	*K =* − 3.766 *B_1_* = 0.544 *B_2_* = − 3.899 *B_3_* = 0.449

This table presents the regression coefficients and *R*^2^ gains for predictive models of receptive and expressive language outcomes at 24 months, based on variables assessed at 12 months. The models include global and MLPT-specific samples, with variables such as social communication behaviors, language and cognitive abilities, and sociodemographic factors contributing to the explained variance. Candidate variables not listed were excluded from the model due to non-significant contributions (*p* > 0.05). Entered variables are reported in the order of entry, i.e., from the one with the highest predictive weight to the one with the lowest. *Adjusted *R*^2^ = adjusted R-squared. All coefficients were significant at *p* < 0.05. *K* = model constant; *B* = unstandardized coefficients of the model.

For the global model of receptive language (Model 1), the initial set of predictors included: group (MLPT/ FT), GA, birth weight, pregnancy-related complications, neonatal complications, SES; Cognitive and Expressive from the Bayley-III; Expressive and Receptive language, Personal, and ABC from the Vineland-3; Pointing, Eye contact, Waving ‘bye-bye’, Imitation, Response to name, Follows point, Says 1–3 clear words, Understands simple instructions, and Attending to sounds from the SACS-R. To avoid redundancy, the Receptive variable from the Bayley-III at 12 months was excluded. After stepwise estimation, the significant predictors in Model 1 were: Pointing, Cognitive, Attending to sounds, Follows point, and group (MLPT/FT). Together, these predictors explained 50% of the variability in receptive language at 24 months (adjusted *R*^2^ of 0.50).

In the specific model of receptive language (Model 2), the following predictors were considered: SES; Cognitive and Expressive from the Bayley-III; Expressive and Receptive language, Interpersonal, and ABC from the Vineland-3; Pointing, Eye contact, Response to name, Follows point, Understands simple instructions, and Attending to sounds from the SACS-R. The significant predictors in Model 2 were: Attending to sounds, Follows point and Cognitive, which together explain 60.5% of the variability in MLPT infants’ receptive language at 24 months (adjusted *R*^2^ of 0.605).

Regarding the global model of expressive language (Model 3), the following independent variables were entered: GA, pregnancy-related complications, neonatal complications, SES; Cognitive and Receptive from the Bayley-III; Receptive and Expressive language, Interpersonal, and ABC from the Vineland-3; Pointing, Eye contact, Waving ‘bye-bye’, Response to name, Follows point, Says 1–3 clear words, Understands simple instructions, and Attending to sounds from the SACS-R. The Expressive variable from the Bayley-III at 12 months was not included to avoid redundancy. The significant predictors in Model 3 were: Cognitive, Pointing and Interpersonal, which together explain 38% of the variability in expressive language at 24 months (adjusted *R*^2^ of 0.380).

Finally, for the specific model of expressive language (Model 4), the following predictors were considered: SES; Cognitive and Receptive from the Bayley-III; Receptive and Expressive language, Interpersonal, and ABC from the Vineland-3; Pointing, Response to name, and Attending to sounds from the SACS-R. The significant predictors in Model 4 were: Interpersonal, Attending to sounds and Receptive, which together explain 37.2% of the variability in MLPT infants’ expressive language at 24 months (adjusted *R*^2^ = 0.372).

## 4 Discussion

### 4.1 Longitudinal assessment of early developmental differences between MLPT and FT

For early developmental assessment, the cognitive and language subscales of the Bayley-III were used, along with the subdomains of the Vineland-3 to evaluate adaptive behavior, and the SACS-R to screen early attentional and social communication skills. Regarding the Bayley-III, infants consistently demonstrated better performance on the cognitive scale than on both language subscales across the three assessment time points. At 12 months, significant differences were observed in Cognitive, with MLPT infants scoring lower than their FT peers, aligning with previous findings (Oliveira et al., 2024; Sansavini et al., 2014). At this age, no significant differences were observed in Receptive or Expressive, consistent with Benassi et al. (2016) findings on extremely preterm infants. The relatively low linguistic demands at this age, combined with potential limitations of the Bayley-III in detecting subtle language variations, may obscure or underestimate early delays, as suggested by studies on preterm populations (Spencer-Smith et al., 2015; Garfinkle et al., 2024). At 18 months, difficulties in Receptive became apparent. Similar results were reported by Greene et al. (2013) in very low birth weight infants, highlighting that receptive language exhibited the most pronounced differences among subscales, with preterm infants demonstrating significantly lower scores over time. Finally, by 24 months, significant group differences were observed across all Bayley-III scales. These findings align with those of Olsen et al. (2022), who reported similar differences in extremely preterm infants, suggesting that developmental disparities are also evident in MLPT infants. Conversely, parental ratings on the Vineland-3 showed no significant group differences across the three assessment time points, in contrast to the objectively measured differences identified through the Bayley-III. In some subdomains, such as Expressive language and Personal, MLPT infants even scored higher. These results may reflect parental overestimation of their infants’ abilities or an adjustment of their expectations to accommodate early developmental challenges (Sansavini et al., 2011). The lack of significant group differences in adaptive measures underscores the limitations of parent-reported tools, which may fail to accurately capture developmental delays, particularly in MLPT infants. Finally, although a higher proportion of MLPT infants were at risk for delays compared to FT, the SACS-R did not reveal significant group differences. T This finding contradicts our expectations based on ESCS results at 12 months in extremely and very preterm infants (Mateus et al., 2019; De Schuymer et al., 2011; Olafsen et al., 2006). The developmental surveillance approach and categorical nature of the SACS-R, primarily designed to identify autism-related behaviors, may limit its sensitivity in detecting group differences in research contexts. Nonetheless, the SACS-R’s capacity to flag early social communication challenges, which may also indicate broader developmental risks such as language delays (Barbaro and Dissanayake, 2010), underscores its value as a tool for prevention and early intervention.

### 4.2 Interactions between group and assessment time points with Bayley-III

Main effects of group and assessment time point were significant across all Bayley-III scales, indicating that MLPT infants consistently underperformed compared to term peers, and that overall performance varied significantly across time points. Interaction effects between group and assessment time points were not significant for Cognitive. However, a marginally significant effect was found for Expressive, indicating that the gap may start to widen between 18 and 24 months. Finally, a significant interaction effect was observed for Receptive, emerging at 18 months and persisting at 24 months. This suggest a widening developmental gap in language domains over time, that aligns with prior evidence showing that cognitive delays in preterm infants tend to remain stable but may contribute to language-specific deficits (Zimmerman, 2018). Receptive difficulties may precede and intensify expressive language delays, underscoring the foundational role of comprehension in supporting language production. As environmental demands become more linguistically and cognitively complex, deficits in comprehension can further exacerbate challenges in cognitive development, given that language serves as a critical tool for navigating and processing increasingly intricate social and cognitive tasks (Guarini et al., 2016; Sansavini et al., 2014). This brings into consideration when catch-up occurs, as corrected age is commonly used until 2 years of age (Parekh et al., 2016) and often discontinued thereafter, assuming that preterm infants have closed the gap with their FT peers. However, there is increasing uncertainty about when to discontinue age correction. In line with this, our findings suggest that MLPT infants continue to experience cumulative difficulties as developmental demands increase, indicating that the trajectory of their development remains distinct beyond this point. Prior research supports this pattern, showing that differences across all Bayley-III scales persist beyond 36 months in preterm infants, even when corrected age is applied (Sansavini et al., 2014; López-Hernández et al., 2021). Greene et al. (2013) further reported that delays in cognitive and receptive language skills among very low birth weight preterm infants become more pronounced between the first and second years of life. Similarly, Zimmerman (2018) highlights persistent deficits in both expressive and receptive language that extend into the school years among very preterm children. These findings emphasize the complex and non-linear nature of early social communication, cognitive development, and language acquisition in preterm population, reinforcing the need for sustained and targeted intervention strategies strategies that extend beyond infancy to effectively support their developmental progress.

### 4.3 Predictive models of 24-month language outcomes on the Bayley-III

Predictors of language outcomes at 24 months were identified through early social communication behaviors assessed at 12 months using the SACS-R, alongside developmental measures from the Bayley-III, parent-reported subdomains of the Vineland-3, and clinical and sociodemographic factors. The SACS-R effectively identified early social communication behaviors that predicted Receptive and Expressive performance at 24 months.

Previous research has consistently demonstrated the link between early social communication skills and later language performance. For example, Suttora and Salerni (2012) reported that communicative gestures, such as pointing at 12 months, are positively associated with linguistic skills at 18 and 24 months in very preterm infants. Gestures, particularly pointing, facilitate interaction and shared meaning, which are fundamental for language learning (Olafsen et al., 2006). Similarly, Wong et al. (2014) found that higher scores on autism-related checklists, such as the Q-CHAT, are associated with lower Bayley-III language scores in extremely and very preterm infants. While these behaviors are often assessed in autism screening, their importance for joint attention and social interaction makes them central to early language development. In our study, key predictors of language outcomes at 24 months included Attending to sounds, Follows point, and Pointing, with better performance on these social communication behaviors at 12 months being positively associated with higher Receptive and Expressive scores at 24 months. These items represent the core attentional processes and joint attention skills assessed by the SACS-R, highlighting their central role in early language development. In line with this, Di Rosa et al. (2016) found that extremely preterm infants at 24 months showed persistent developmental delays, with attention and language issues being prominent outcomes. In the receptive language models, attentional response behaviors (e.g., Attending to sounds and Follows point) were significant predictors in both the global and preterm-specific models. This reinforces the role of early attentional regulation in supporting receptive language acquisition, as these behaviors require the infant to focus and respond to meaningful social stimuli. Furthermore, the initiating joint attention behavior of Pointing emerged as a key predictor of both Receptive and Expressive in the global models. This emphasizes the universal importance of pointing for language performance, regardless of GA. Suttora and Salerni (2012) identified pointing as a significant skill in very preterm infants, whereas De Schuymer et al. (2011) highlighted initiating behavioral requests as the strongest predictor of expressive language development in the same population. Early social communication behaviors that rely on attentional processes are closely linked to broader cognitive functions. The ability to sustain and direct attention toward relevant stimuli facilitates early engagement in social interactions, which forms the foundation for receptive and expressive language growth.

Additionally, Cognitive at 12 months emerged as significant predictors of Receptive performance at 24 months, whereas Receptive at 12 months predicted Expressive performance at 24 months, in both the global and preterm-specific models. Once again, cognitive skills may provide the foundation for early comprehension, and comprehension, in turn, facilitates expressive language growth. Finally, in both expressive language models, Interpersonal skills was the only subdomain of the Vineland-3 to emerge as a significant predictor. Higher performance in Interpersonal skills at 12 months was associated with better Expressive performance at 24 months, reflecting the broader role of early social abilities in supporting expressive language development.

Regarding other potential predictors described in the literature, birth status (MLPT/FT) was a significant predictor of Receptive performance, consistent with De Schuymer et al. (2011), with MLPT infants showing a higher risk for delays. In contrast, sociodemographic and neonatal factors, such as SES and clinical complications, were not significant predictors in our models. This aligns with other studies on preterm populations, which reported minimal influence of SES, birth weight or GA on language outcomes (Van Noort-van Der Spek et al., 2012; Di Rosa et al., 2016). However, other investigations found strong associations between language outcomes and factors such as socioeconomic disadvantage (e.g., SES, ethnicity) and neonatal risks (Greene et al., 2013; Wong et al., 2014; Palomo-Osuna et al., 2022). These discrepancies underscore the need for further research using adequate methodologies to better understand the role of SES, clinical factors, and environmental influences.

Overall, this study highlights the utility of tools like the SACS-R in identifying early social communication deficits that predict later language delays, particularly in Receptive, which consistently emerged as the most affected domain across all analyses. While primarily designed for autism screening, our findings demonstrate its broader value in detecting risks for language development. Given the foundational role of receptive in supporting expressive language and overall developmental outcomes, integrating tools like the SACS-R into early screening protocols could facilitate timely interventions that target both social communication skills and language outcomes. This, in turn, could support better developmental trajectories for at-risk populations, such as MLPT.

### 4.4 Factors influencing development and future research directions

Finally, several considerations should be taken into account regarding this study. One key limitation is the use of convenience sampling, combined with differences in recruitment methods between MLPT and FT groups, may limit the generalizability of the findings. However, this approach is common in studies of this nature due to the challenges of accessing this specific population.

One important consideration is that cognitive performance was not statistically controlled as a covariate, as it was treated as a variable of interest in this study. Consequently, it remains unclear to what extent the observed effects and predictions would hold if cognitive performance had been accounted for (Van Noort-van Der Spek et al., 2012; Zimmerman, 2018). This limitation may be particularly relevant in Model 1, where cognitive performance plays a significant role, and other explanatory variables could potentially better account for receptive performance. However, at the descriptive level, although a portion of MLPT infants scored below the standard cutoff of 85 on the Bayley-III cognitive scale across all assessment points, a consistently higher proportion fell below this threshold on the language subscales. This pattern suggests that some language delays may extend beyond general cognitive challenges. Future studies should control for cognitive scores for the generalizability of conclusions.

Another relevant aspect is the potential influence of interventions received during the first 2 years on language development was not examined. While much of the literature on early development focuses on how parental emotional states (Muller-Nix et al., 2004; Treyvaud et al., 2010; Gueron-Sela et al., 2015; Feldman and Eidelman, 2007) and interaction styles (Delonis et al., 2017; Korja et al., 2010; Hall et al., 2015; Sansavini et al., 2015; Loi et al., 2017) influence the development of their preterm infants, interventions initiated in the NICU (i.e., effective touch through skin-to-skin contact or kangaroo care) (La Rosa et al., 2024; Lejeune et al., 2019; Beltrán et al., 2022), or studies directly implementing early interventions (Bejarano-Martín et al., 2020; Meijssen et al., 2010; Wu et al., 2016), less attention has been given to research that considers early interventions as a study variable, particularly those targeting the development of specific domains. This highlights the need for further research in this area, as it is directly related to the developmental outcomes of preterm infants, the catch-up process of their FT peers, and how they close the gap.

Lastly, the cohort examined in this study was born during the COVID-19 pandemic, a factor that has been linked to reduced social interaction variability—due to restrictions and mask usage—which in turn has been associated with negative effects on language development (Feijoo et al., 2023). However, specific pandemic-related factors were not included in the analyses.

These considerations provide context for interpreting the findings and highlight areas for potential future investigation into the developmental trajectories and challenges faced by MLPT infants.

## Data Availability

The raw data supporting the conclusions of this article will be made available by the authors, without undue reservation.
