# Supplementary material for: Early social communication and language development in moderate-to-late preterm infants: a longitudinal study
Source: Front Psychol. 2025 Apr 3;16:1556416. doi: 10.3389/fpsyg.2025.1556416 (PMC12003367; doi:10.3389/fpsyg.2025.1556416)
Supplement: Supplementary file 1 [file Table_1.pdf]

Supplementary Table 1.

Correlation matrix between 12-month measures and Receptive and Expressive at 24 months

	Global (FT + MLPT infants)		MLPT infants	
**Constant variables (12 months)**	**Receptive at 24 months**	**Expressive at 24 months**	**Receptive at 24 months**	**Expressive at 24 months**
Group[a]	**-.391****	**-.279****	-	-
GA	**.381****	**.264***	.134	.106
Birth weight	**.296****	.198	.053	.006
Pregnancy-related complications[a]	**-.315****	**-.308****	-.142	-.076
Neonatal complications[a]	**-.327****	**-.274***	-.155	-.091
Mother's age at conception	-.099	-.066	-.093	-.018
Father's age at conception	-.212	-.158	-.229	-.143
SES	**.272***	**.251***	**.360***	**.388***
**Bayley-III**				
Cognitive	**.474****	**.487****	**.439****	**.433****
Receptive language	**.473****	**.549****	**.386***	**.428****
Expressive language	**.267***	**.311****	**.313***	.271
**Vineland-3**				
Receptive language	**.269***	**.275***	**.423****	**.316***
Expressive language	.202	**.253***	**.432****	**.395***
Personal	**.215***	.158	.213	.097
Interpersonal	.177	**.310****	**.479****	**.498****
Play and leisure	.173	.123	.266	.135
Adaptative Behavior Composite	**.287****	**.301****	**.424****	**.333***
**SACS-R**[a]				
Pointing	**-.442****	**-.433****	**-.434****	**-.427****
Eye contact	**-.273***	**-.245***	**-.339***	-.233
Waving 'bye-bye'	**-.313****	**-.269***	-.306	-.195
Imitation	**-.240***	-.175	-.278	-.184
Response to name	**-.279****	**-.249***	**-.416****	**-.359***
Follows point	**-.325****	**-.311****	**-.430****	-.243
Social smile	-.032	-.055	-.109	-.043
Conversational babble	-.108	-.167	-.057	-.189
Says 1-3 clear words	**-.294****	**-.252***	-.195	-.217
Understands simple instructions	**-.449****	**-.344****	**-.443****	-.291
Attending to sounds	**-.311****	**-.257***	**-.540****	**-.469****

Note. * $p < .05$; ** $p < .01$. [a] Categorical variables.
